# Supplementary material for: Escalation Time to Open Triple Combination Therapy from the Initiation of LAMA versus ICS/LABA in COPD Management: Findings from Comparing the Incidence of Tiotropium and ICS/LABA in Real-World Use in South Korea (CITRUS) Study
Source: J Pers Med. 2021 Dec 7;11(12):1325. doi: 10.3390/jpm11121325 (PMC8703927; doi:10.3390/jpm11121325)
Supplement: Supplementary file 1 [file jpm-11-01325-s001.zip › jpm-1446922-supplementary.pdf]

Journal of Personalized Medicine

MDPI

Supplementary Material: Escalation Time to Open Triple Combination Therapy from the Initiation of LAMA versus ICS/LABA in COPD Management: Findings from Comparing the Incidence of Tiotropium and ICS/LABA in Real-World Use in South Korea (CITRUS) Study

Ye Jin Lee, Chin Kook Rhee, Yong Il Hwang, Kwang Ha Yoo, So Eun Lee, Doik Lee, Yong Bum Park and Youlim Kim

Table S1. Baseline characteristics of the crude population.

Baseline Characteristics	All (*n* = 9284)	LAMA (*n* = 6352)	ICS/LABA (*n* = 2932)	*p* Value
Observational period, days	764.7 ± 636.8	787.0 ± 639.5	716.5 ± 628.4	<0.001
Age, years	69.8 ± 7.8	69.89 ± 7.8	69.59 ± 7.9	0.09
55 to <65, n (%)	2530 (27)	1690 (27)	840 (29)	0.11
65 to <75, n (%)	4068 (44)	2800 (44)	1268 (43)	
75+, n (%)	2686 (29)	1862 (29)	824 (28)	
Sex, male	7545 (81)	5394 (85)	2151 (73)	0.001
Income level				
1st quartile, n (%)	1385 (15)	947 (15)	438 (15)	<0.001
2nd quartile, n (%)	1287 (14)	926 (15)	361 (12)	
3rd quartile, n (%)	1923 (21)	1391 (22)	532 (18)	
4th quartile, n (%)	3194 (34)	2301 (36)	893 (30)	
Medical aid, n (%)	1495 (16)	787 (12)	708 (24)	
History of COPD exacerbation				
None, n (%)	7207 (78)	4987 (79)	2220 (76)	0.01
1 moderate, n (%)	833 (9)	559 (9)	274 (9)	
≥2 moderate OR ≥ 1 severe, n (%)	1244 (13)	806 (13)	438 (15)	
History of asthma				
No, n (%)	5599 (60)	4021 (63)	1578 (54)	0.00
Yes, n (%)	3685 (40)	2331 (37)	1354 (46)	
History of pneumonia				
No, n (%)	8208 (88)	5548 (87)	2660 (91)	0.00
Yes, n (%)	1076 (12)	804 (13)	272 (9)	
mCCI	1.95 ± 1.87	2.01 ± 1.89	1.82 ± 1.81	0.00

Data are expressed as mean ± standard deviation or *n* (%).LAMA, long-acting muscarinic antagonist; ICS/LABA, inhaled corticosteroid plus long-acting beta-2 agonist; COPD, chronic obstructive pulmonary disease; mCCI, modified Charlson Comorbidity index.

Journal of Personalized Medicine

MDPI

Table S2. Hazard ratios of triple combination therapy in the propensity score-matched population (LAMA as reference).

Variables		Event		HR		
		n	%	HR	95% CI	*p* Value
**Overall**		901	0.18	1.601	1.404–1.826	<0.001
Age (years)	55 to <65	277	0.2	1.392	1.097–1.766	0.006
	65 to <75	400	0.19	1.761	1.442–2.152	<0.001
	75+	224	0.16	1.630	1.249–2.127	0.001
p for interaction = 0.296						
Sex	Male	73	0.2	1.747	1.511–2.022	<0.001
	Female	148	0.12	1.100	0.795–1.522	0.566
p for interaction = 0.016						
History of COPD exacerbation	None	637	0.17	1.619	1.382–1.896	<0.001
	1 moderate	104	0.21	1.733	1.174–2.558	0.006
	≥2 moderate OR ≥1 severe	160	0.21	1.438	1.052–1.966	0.023
p for interaction = 0.801						
History of asthma	No	438	0.17	1.793	1.480–2.171	<0.001
	Yes	463	0.20	1.436	1.194–1.726	0.001
p for interaction = 0.104						
History of pneumonia	No	807	0.18	1.630	1.416–1.876	<0.001
	Yes	84	0.18	1.381	0.920–2.072	0.119
p for interaction = 0.445						

Data are expressed as mean ± standard deviation or *n* (%). LAMA, long-acting muscarinic antagonist; ICS/LABA, inhaled corticosteroid plus long-acting beta-2 agonist; COPD, chronic obstructive pulmonary disease.
